# Diisoprop­yl{2-[2-(2-oxopyrrolidin-1-yl)acetamido]eth­yl}ammonium hydrogen sulfate

**DOI:** 10.1107/S1600536808015341

**Published:** 2008-05-30

**Authors:** Massimo Bambagiotti-Alberti, Gianluca Bartolucci, Bruno Bruni, Silvia A. Coran, Massimo Di Vaira

**Affiliations:** a Dipartimento di Scienze Farmaceutiche, Universitá di Firenze, Via U. Schiff 6, I-50019 Sesto Fiorentino, Firenze, Italy; b Dipartimento di Chimica, Universitá di Firenze, Via della Lastruccia 3, I-50019 Sesto Fiorentino, Firenze, Italy

## Abstract

The structure of the title compound, C_14_H_28_N_3_O_2_^+^·HSO_4_^−^, a nootropic drug (pramiracetam) investigated for cognition-enhancing properties, is closely similar to that of the previously determined acetonitrile solvate, both structures being characterized by the presence of ribbons of hydrogen-bonded ions. The pyrrolidine ring adopts an envelope conformation.

## Related literature

For related literature, see: Claus *et al.* (1991 ▶); Gouliaev & Senning (1994 ▶); Mondadori *et al.* (1991 ▶); Pugsley *et al.* (1983 ▶). For a related structure, see: Bandoli *et al.* (1987 ▶).
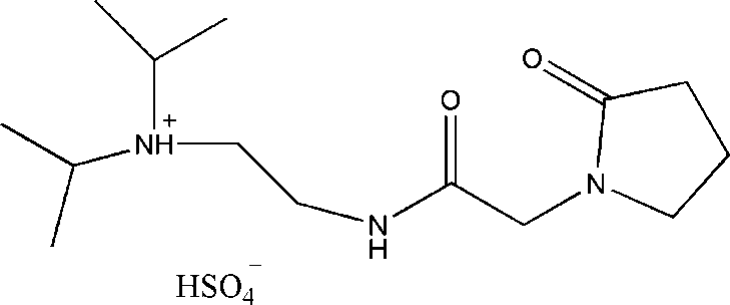


## Experimental

### 

#### Crystal data

C_14_H_28_N_3_O_2_^+^·HSO_4_^−^
*M* *_r_* = 367.46
Triclinic, 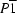

*a* = 6.7834 (3) Å
*b* = 11.1755 (4) Å
*c* = 12.9012 (6) Å
α = 72.165 (4)°
β = 89.394 (4)°
γ = 89.509 (3)°
*V* = 930.95 (7) Å^3^
*Z* = 2
Cu *K*α radiation
μ = 1.85 mm^−1^
*T* = 170 (2) K
0.50 × 0.25 × 0.10 mm

#### Data collection

Oxford Diffraction Xcalibur PX Ultra CCD diffractometer
Absorption correction: multi-scan (*ABSPACK* in *CrysAlisPro RED*; Oxford Diffraction, 2006 ▶) *T*_min_ = 0.762, *T*_max_ = 1.000 (expected range = 0.634–0.831)
7656 measured reflections
3356 independent reflections
3253 reflections with *I* > 2σ(*I*)
*R*_int_ = 0.018

#### Refinement

*R*[*F* ^2^ > 2σ(*F* ^2^)] = 0.046
*wR*(*F* ^2^) = 0.132
*S* = 1.06
3356 reflections
230 parameters
H atoms treated by a mixture of independent and constrained refinement
Δρ_max_ = 0.45 e Å^−3^
Δρ_min_ = −0.57 e Å^−3^

### 

Data collection: *CrysAlisPro CCD* (Oxford Diffraction, 2006 ▶); cell refinement: *CrysAlisPro CCD*; data reduction: *CrysAlisPro RED* (Oxford Diffraction, 2006 ▶); program(s) used to solve structure: *SIR97* (Altomare *et al.*, 1999 ▶); program(s) used to refine structure: *SHELXL97* (Sheldrick, 2008 ▶); molecular graphics: *ORTEP-3* (Farrugia, 1997 ▶) and *PLATON* (Spek, 2003 ▶); software used to prepare material for publication: *SHELXL97*.

## Supplementary Material

Crystal structure: contains datablocks global, I. DOI: 10.1107/S1600536808015341/pv2084sup1.cif

Structure factors: contains datablocks I. DOI: 10.1107/S1600536808015341/pv2084Isup2.hkl

Additional supplementary materials:  crystallographic information; 3D view; checkCIF report

## Figures and Tables

**Table 1:** Hydrogen-bond geometry (Å, °)

*D*—H⋯*A*	*D*—H	H⋯*A*	*D*⋯*A*	*D*—H⋯*A*
N2—H2⋯O3	0.86 (3)	2.10 (3)	2.948 (2)	172 (2)
N3—H3⋯O4^i^	0.97 (3)	1.79 (3)	2.764 (2)	175 (2)
O6—H6⋯O1^ii^	0.90 (4)	1.68 (4)	2.559 (2)	167 (3)
C12—H12⋯O2^iii^	1.00	2.40	3.362 (3)	162
C13—H131⋯O4^iv^	0.98	2.59	3.559 (2)	171
C14—H143⋯O5	0.98	2.56	3.530 (2)	172

Symmetry codes: (i) 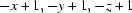; (ii) 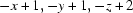; (iii) 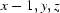; (iv) 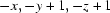.

**Fig. 1.**
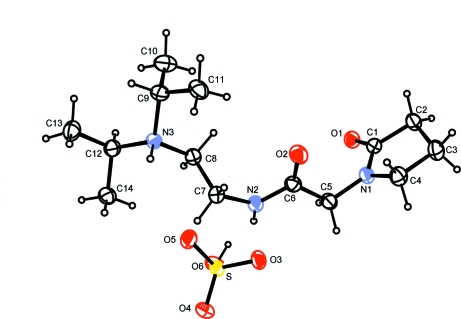
A view of the content of the asymmetric unit of (I). Displacement ellipsoids are drawn at the 50% probability level.

**Fig. 2.**
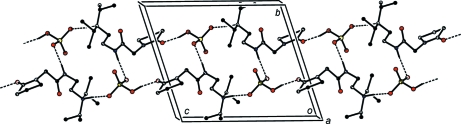
A view of the crystal packing in the structure of (I), showing the presence of ribbons formed by hydrogen–bonded ions. Hydrogen bonds are denoted by dashed lines. Only hydrogen atoms involved in the formation of hydrogen bonds are shown.

## supplementary crystallographic information

### Comment

Pramiracetam is a nootropic drug (Gouliaev & Senning, 1994) whose neurochemical properties have been investigated (Pugsley *et al.*, 1983) also in view of possible applications as a memory aid (Mondadori *et al.*, 1991) and for symptomatic treatment of Alzheimer's desease (Claus *et al.*, 1991).

The contents of the asymmetric unit of the title compound (I) are shown in Fig 1. The atomic labelling is consistent with that previously adopted for the CH_3_CN solvate (Bandoli *et al.*, 1987) with which (I) is isomorphous and substantially isostructural, although the present conventional choice of labels for the axes does not match that of the previous report. There is a 3.8% reduction in the cell volume going from the solvated form to the present one, due to contraction of the two longest cell axes. The packing (Fig. 2) is controlled by a network of hydrogen bonds. The HSO_4_^-^ anion behaves as a hydrogen bond donor towards a carbonylic oxygen and acts as acceptor of two hydrogen bonds from the aminic and ammonium N atoms (details of hydrogen-bonding geometry have been provided in Table 1). In this way two centrosymmetric and adjacent loops, consisting of 14 and 18 non-hydrogen atoms, respectively, are formed. Each loop has contributions from parts of two anions and two cations and each of these ions contributes to two contiguous loops which share the N2—H2···O3—S strand. Such arrangement, combined with the operation of the c translation, generates ribbons in the structure (these were parallel to the a direction in the previously reported structure of the solvated form, due to the different labelling of cell axes). In the case of the CH_3_CN solvate the solvent molecules occupy large voids among the ribbons and are not involved in the network of hydrogen bonds. The largest difference in the conformation of the cation between the two structures is found for the C1—N1—C5—C6 torsion angle, whose value of -94.6 (2)° for (I) is significantly smaller than that, of -105.4(1.2)°, found for the structure of the solvate. The structure of (I) is stabilized by non-classical intermolecular and intramolecular hydrogen bonds of the type C—H···O (Table 1).

### Experimental

Samples of pramiracetam were kindly provided by SIMS (SIMS srl, Reggello Firenze, Italy). Crystals of (I), suitable for X-ray diffraction analysis, were obtained by slow evaporation from 1:3 2-propanol:acetone solutions.

### Refinement

A small fraction of data is missing from completeness because the structure appeared to be monoclinic at the time of data collection. It was impossible to collect a new set of data due to extreme difficulty to obtain suitable material for diffraction. Also, since crystals did not diffract strongly, it was deemed that collecting data at θ higher than 72° would not yield significant improvement. H atoms bound to carbon atoms were in geometrically generated positions, riding; the coordinates of those bound to N and O atoms were refined freely. The constraint *U(H)* = 1.2*U*_eq_(C,*N*) was applied [*U(H)* = 1.5*U*_eq_(C) for methyl group H atoms]. Bond distances involving refined hydrogen atoms: N2—H2 0.86 (3) Å, N3—H3 0.97 (3) Å, O6—H6 0.90 (4) Å. The only residual electron density of any numerical, but not chemical significance is in close proximity of the O and S atoms of the counterion. The existence of voids (57.0 Å^3^) in the structure is likely due to the stability of the hydrogen-bonds framework, as the solvents used for crystallization could not fit into the available spacing.

### Crystal data

**Table:**

C_14_H_28_N_3_O_2_^+^·HSO_4_^–^	*Z* = 2
*M**_r_* = 367.46	*F*_000_ = 396
Triclinic, *P*1	*D*_x_ = 1.311 Mg m^−^^3^
Hall symbol: -P 1	Cu *K*α radiation λ = 1.54180 Å
*a* = 6.7834 (3) Å	Cell parameters from 6722 reflections
*b* = 11.1755 (4) Å	θ = 4.6–72.4º
*c* = 12.9012 (6) Å	µ = 1.85 mm^−^^1^
α = 72.165 (4)º	*T* = 170 (2) K
β = 89.394 (4)º	Prism, pale yellow
γ = 89.509 (3)º	0.50 × 0.25 × 0.10 mm
*V* = 930.95 (7) Å^3^	

### Data collection

**Table:**

Oxford Diffraction Xcalibur PX Ultra CCD diffractometer	3356 independent reflections
Radiation source: fine-focus sealed tube	3253 reflections with *I* > 2σ(*I*)
Monochromator: Oxford Diffraction Enhance ULTRA assembly	*R*_int_ = 0.018
Detector resolution: 8.1241 pixels mm^-1^	θ_max_ = 72.7º
*T* = 170(2) K	θ_min_ = 4.6º
ω scans	*h* = −8→8
Absorption correction: multi-scan(ABSPACK in CrysAlisPro RED; Oxford Diffraction, 2006)	*k* = −13→13
*T*_min_ = 0.762, *T*_max_ = 1.000	*l* = −15→15
7656 measured reflections	

### Refinement

**Table:**

Refinement on *F*^2^	Secondary atom site location: difference Fourier map
Least-squares matrix: full	Hydrogen site location: inferred from neighbouring sites
*R*[*F*^2^ > 2σ(*F*^2^)] = 0.046	H atoms treated by a mixture of independent and constrained refinement
*wR*(*F*^2^) = 0.132	*w* = 1/[σ^2^(*F*_o_^2^) + (0.0779*P*)^2^ + 0.7081*P*] where *P* = (*F*_o_^2^ + 2*F*_c_^2^)/3
*S* = 1.06	(Δ/σ)_max_ < 0.001
3356 reflections	Δρ_max_ = 0.45 e Å^−^^3^
230 parameters	Δρ_min_ = −0.57 e Å^−^^3^
Primary atom site location: structure-invariant direct methods	Extinction correction: none

### Special details

**Table:**

Geometry. All e.s.d.'s (except the e.s.d. in the dihedral angle between two l.s. planes) are estimated using the full covariance matrix. The cell e.s.d.'s are taken into account individually in the estimation of e.s.d.'s in distances, angles and torsion angles; correlations between e.s.d.'s in cell parameters are only used when they are defined by crystal symmetry. An approximate (isotropic) treatment of cell e.s.d.'s is used for estimating e.s.d.'s involving l.s. planes.
Refinement. Refinement of *F*^2^ against ALL reflections. The weighted *R*-factor *wR* and goodness of fit *S* are based on *F*^2^, conventional *R*-factors *R* are based on *F*, with *F* set to zero for negative *F*^2^. The threshold expression of *F*^2^ > σ(*F*^2^) is used only for calculating *R*-factors(gt) *etc*. and is not relevant to the choice of reflections for refinement. *R*-factors based on *F*^2^ are statistically about twice as large as those based on *F*, and *R*- factors based on ALL data will be even larger.

### Fractional atomic coordinates and isotropic or equivalent isotropic displacement parameters (Å^2^)

**Table:**

	*x*	*y*	*z*	*U*_iso_*/*U*_eq_	
S	0.38398 (7)	0.68601 (4)	0.65914 (4)	0.02428 (17)	
O3	0.5651 (2)	0.65687 (14)	0.72078 (13)	0.0327 (4)	
O4	0.4059 (2)	0.78663 (14)	0.55696 (12)	0.0308 (3)	
O5	0.2944 (2)	0.57503 (15)	0.64603 (14)	0.0386 (4)	
O6	0.2374 (3)	0.74531 (16)	0.72349 (14)	0.0384 (4)	
H6	0.234 (5)	0.703 (3)	0.795 (3)	0.058*	
N1	1.0351 (2)	0.37637 (16)	0.93852 (14)	0.0254 (4)	
C1	0.9684 (3)	0.33125 (18)	1.04001 (16)	0.0243 (4)	
O1	0.7977 (2)	0.34687 (14)	1.06959 (12)	0.0289 (3)	
C2	1.1312 (3)	0.2564 (2)	1.11073 (17)	0.0287 (4)	
H21	1.1096	0.1649	1.1271	0.034*	
H22	1.1402	0.2768	1.1800	0.034*	
C3	1.3171 (3)	0.2979 (2)	1.04077 (18)	0.0328 (5)	
H31	1.3827	0.3679	1.0584	0.039*	
H32	1.4116	0.2272	1.0515	0.039*	
C4	1.2393 (3)	0.3403 (2)	0.92434 (18)	0.0310 (5)	
H41	1.3151	0.4126	0.8779	0.037*	
H42	1.2451	0.2710	0.8916	0.037*	
C5	0.9126 (3)	0.44164 (18)	0.84733 (17)	0.0267 (4)	
H51	0.9903	0.5092	0.7960	0.032*	
H52	0.8002	0.4815	0.8740	0.032*	
C6	0.8339 (3)	0.35436 (18)	0.78717 (16)	0.0254 (4)	
O2	0.8971 (2)	0.24667 (13)	0.80310 (13)	0.0322 (4)	
N2	0.6967 (3)	0.40757 (16)	0.71317 (14)	0.0268 (4)	
H2	0.649 (4)	0.479 (3)	0.711 (2)	0.032*	
C7	0.6169 (3)	0.34383 (19)	0.64051 (17)	0.0268 (4)	
H71	0.7204	0.2906	0.6220	0.032*	
H72	0.5733	0.4067	0.5722	0.032*	
C8	0.4429 (3)	0.26239 (18)	0.69493 (16)	0.0254 (4)	
H81	0.4831	0.2079	0.7678	0.031*	
H82	0.3340	0.3173	0.7052	0.031*	
N3	0.3696 (2)	0.18130 (15)	0.62949 (14)	0.0236 (4)	
H3	0.449 (4)	0.197 (2)	0.564 (2)	0.028*	
C9	0.3977 (3)	0.04104 (18)	0.69036 (18)	0.0289 (4)	
H9	0.3453	−0.0071	0.6429	0.035*	
C10	0.2806 (4)	0.0020 (2)	0.7957 (2)	0.0398 (5)	
H101	0.1418	0.0254	0.7806	0.060*	
H102	0.3327	0.0445	0.8456	0.060*	
H103	0.2914	−0.0892	0.8291	0.060*	
C11	0.6165 (3)	0.0111 (2)	0.70570 (19)	0.0342 (5)	
H111	0.6351	−0.0801	0.7359	0.051*	
H112	0.6708	0.0525	0.7559	0.051*	
H113	0.6847	0.0415	0.6352	0.051*	
C12	0.1589 (3)	0.21577 (18)	0.58962 (17)	0.0264 (4)	
H12	0.0700	0.2061	0.6540	0.032*	
C13	0.0884 (3)	0.1305 (2)	0.5264 (2)	0.0341 (5)	
H131	−0.0412	0.1593	0.4953	0.051*	
H132	0.0780	0.0443	0.5754	0.051*	
H133	0.1825	0.1328	0.4677	0.051*	
C14	0.1534 (3)	0.35207 (19)	0.51788 (18)	0.0297 (4)	
H141	0.0187	0.3748	0.4923	0.045*	
H142	0.2418	0.3626	0.4552	0.045*	
H143	0.1963	0.4067	0.5599	0.045*	

### Atomic displacement parameters (Å^2^)

**Table:**

	*U*^11^	*U*^22^	*U*^33^	*U*^12^	*U*^13^	*U*^23^
S	0.0230 (3)	0.0252 (3)	0.0239 (3)	0.00295 (18)	−0.0020 (2)	−0.00645 (19)
O3	0.0279 (8)	0.0345 (8)	0.0357 (8)	0.0053 (6)	−0.0098 (7)	−0.0106 (6)
O4	0.0319 (7)	0.0303 (7)	0.0256 (8)	0.0063 (6)	0.0024 (6)	−0.0022 (6)
O5	0.0385 (9)	0.0306 (8)	0.0474 (10)	−0.0018 (6)	−0.0117 (7)	−0.0125 (7)
O6	0.0424 (9)	0.0405 (9)	0.0283 (8)	0.0153 (7)	0.0044 (7)	−0.0053 (7)
N1	0.0230 (8)	0.0282 (8)	0.0241 (9)	0.0020 (6)	−0.0028 (7)	−0.0067 (7)
C1	0.0241 (9)	0.0234 (9)	0.0252 (10)	−0.0011 (7)	−0.0019 (8)	−0.0072 (7)
O1	0.0231 (7)	0.0332 (8)	0.0289 (8)	0.0015 (6)	0.0000 (6)	−0.0076 (6)
C2	0.0264 (10)	0.0312 (10)	0.0266 (10)	0.0037 (8)	−0.0050 (8)	−0.0062 (8)
C3	0.0243 (10)	0.0404 (12)	0.0331 (12)	0.0042 (8)	−0.0041 (9)	−0.0104 (9)
C4	0.0248 (10)	0.0379 (11)	0.0294 (11)	0.0019 (8)	0.0013 (9)	−0.0091 (9)
C5	0.0295 (10)	0.0252 (9)	0.0244 (10)	0.0024 (8)	−0.0043 (8)	−0.0063 (8)
C6	0.0258 (9)	0.0259 (9)	0.0232 (10)	0.0002 (7)	0.0007 (8)	−0.0057 (8)
O2	0.0353 (8)	0.0266 (7)	0.0358 (8)	0.0074 (6)	−0.0075 (7)	−0.0111 (6)
N2	0.0289 (9)	0.0230 (8)	0.0284 (9)	0.0009 (7)	−0.0062 (7)	−0.0073 (7)
C7	0.0274 (10)	0.0274 (10)	0.0254 (10)	−0.0018 (8)	−0.0034 (8)	−0.0076 (8)
C8	0.0256 (9)	0.0260 (9)	0.0246 (10)	0.0001 (7)	−0.0019 (8)	−0.0076 (8)
N3	0.0220 (8)	0.0221 (8)	0.0256 (9)	0.0010 (6)	−0.0015 (7)	−0.0057 (6)
C9	0.0315 (11)	0.0209 (9)	0.0314 (11)	0.0025 (8)	−0.0032 (9)	−0.0038 (8)
C10	0.0436 (13)	0.0301 (11)	0.0390 (13)	0.0002 (9)	0.0036 (11)	−0.0007 (9)
C11	0.0336 (11)	0.0307 (11)	0.0355 (12)	0.0085 (9)	−0.0063 (9)	−0.0062 (9)
C12	0.0213 (9)	0.0265 (10)	0.0304 (11)	0.0009 (7)	−0.0007 (8)	−0.0074 (8)
C13	0.0282 (10)	0.0330 (11)	0.0430 (13)	0.0013 (8)	−0.0073 (9)	−0.0145 (10)
C14	0.0262 (10)	0.0269 (10)	0.0335 (11)	0.0043 (8)	−0.0033 (9)	−0.0056 (8)

### Geometric parameters (Å, °)

**Table:**

S—O5	1.4422 (16)	C7—H71	0.9900
S—O3	1.4504 (14)	C7—H72	0.9900
S—O4	1.4543 (15)	C8—N3	1.505 (2)
S—O6	1.5574 (16)	C8—H81	0.9900
O6—H6	0.90 (4)	C8—H82	0.9900
N1—C1	1.327 (3)	N3—C12	1.530 (2)
N1—C5	1.446 (2)	N3—C9	1.534 (2)
N1—C4	1.465 (3)	N3—H3	0.97 (3)
C1—O1	1.243 (2)	C9—C10	1.512 (3)
C1—C2	1.514 (3)	C9—C11	1.521 (3)
C2—C3	1.533 (3)	C9—H9	1.0000
C2—H21	0.9900	C10—H101	0.9800
C2—H22	0.9900	C10—H102	0.9800
C3—C4	1.528 (3)	C10—H103	0.9800
C3—H31	0.9900	C11—H111	0.9800
C3—H32	0.9900	C11—H112	0.9800
C4—H41	0.9900	C11—H113	0.9800
C4—H42	0.9900	C12—C13	1.514 (3)
C5—C6	1.523 (3)	C12—C14	1.521 (3)
C5—H51	0.9900	C12—H12	1.0000
C5—H52	0.9900	C13—H131	0.9800
C6—O2	1.231 (2)	C13—H132	0.9800
C6—N2	1.339 (3)	C13—H133	0.9800
N2—C7	1.451 (2)	C14—H141	0.9800
N2—H2	0.86 (3)	C14—H142	0.9800
C7—C8	1.525 (3)	C14—H143	0.9800
			
O5—S—O3	111.94 (9)	N3—C8—C7	112.54 (16)
O5—S—O4	112.97 (10)	N3—C8—H81	109.1
O3—S—O4	113.20 (9)	C7—C8—H81	109.1
O5—S—O6	108.27 (10)	N3—C8—H82	109.1
O3—S—O6	107.71 (9)	C7—C8—H82	109.1
O4—S—O6	102.03 (9)	H81—C8—H82	107.8
S—O6—H6	113 (2)	C8—N3—C12	112.04 (15)
C1—N1—C5	123.65 (17)	C8—N3—C9	111.57 (15)
C1—N1—C4	113.69 (16)	C12—N3—C9	113.44 (15)
C5—N1—C4	122.23 (17)	C8—N3—H3	108.8 (14)
O1—C1—N1	124.46 (18)	C12—N3—H3	104.9 (14)
O1—C1—C2	126.64 (18)	C9—N3—H3	105.5 (15)
N1—C1—C2	108.87 (17)	C10—C9—C11	113.40 (19)
C1—C2—C3	103.44 (17)	C10—C9—N3	111.46 (18)
C1—C2—H21	111.1	C11—C9—N3	109.53 (16)
C3—C2—H21	111.1	C10—C9—H9	107.4
C1—C2—H22	111.1	C11—C9—H9	107.4
C3—C2—H22	111.1	N3—C9—H9	107.4
H21—C2—H22	109.0	C9—C10—H101	109.5
C4—C3—C2	103.68 (16)	C9—C10—H102	109.5
C4—C3—H31	111.0	H101—C10—H102	109.5
C2—C3—H31	111.0	C9—C10—H103	109.5
C4—C3—H32	111.0	H101—C10—H103	109.5
C2—C3—H32	111.0	H102—C10—H103	109.5
H31—C3—H32	109.0	C9—C11—H111	109.5
N1—C4—C3	102.97 (17)	C9—C11—H112	109.5
N1—C4—H41	111.2	H111—C11—H112	109.5
C3—C4—H41	111.2	C9—C11—H113	109.5
N1—C4—H42	111.2	H111—C11—H113	109.5
C3—C4—H42	111.2	H112—C11—H113	109.5
H41—C4—H42	109.1	C13—C12—C14	110.05 (18)
N1—C5—C6	112.43 (16)	C13—C12—N3	110.61 (16)
N1—C5—H51	109.1	C14—C12—N3	109.11 (16)
C6—C5—H51	109.1	C13—C12—H12	109.0
N1—C5—H52	109.1	C14—C12—H12	109.0
C6—C5—H52	109.1	N3—C12—H12	109.0
H51—C5—H52	107.9	C12—C13—H131	109.5
O2—C6—N2	123.75 (18)	C12—C13—H132	109.5
O2—C6—C5	122.51 (18)	H131—C13—H132	109.5
N2—C6—C5	113.70 (17)	C12—C13—H133	109.5
C6—N2—C7	122.02 (17)	H131—C13—H133	109.5
C6—N2—H2	119.3 (17)	H132—C13—H133	109.5
C7—N2—H2	118.6 (17)	C12—C14—H141	109.5
N2—C7—C8	109.99 (17)	C12—C14—H142	109.5
N2—C7—H71	109.7	H141—C14—H142	109.5
C8—C7—H71	109.7	C12—C14—H143	109.5
N2—C7—H72	109.7	H141—C14—H143	109.5
C8—C7—H72	109.7	H142—C14—H143	109.5
H71—C7—H72	108.2		
			
C5—N1—C1—O1	−5.0 (3)	O2—C6—N2—C7	−3.3 (3)
C4—N1—C1—O1	−177.65 (18)	C5—C6—N2—C7	174.22 (18)
C5—N1—C1—C2	173.48 (17)	C6—N2—C7—C8	87.1 (2)
C4—N1—C1—C2	0.8 (2)	N2—C7—C8—N3	−172.82 (15)
O1—C1—C2—C3	−165.6 (2)	C7—C8—N3—C12	−115.80 (18)
N1—C1—C2—C3	16.0 (2)	C7—C8—N3—C9	115.77 (18)
C1—C2—C3—C4	−25.4 (2)	C8—N3—C9—C10	62.2 (2)
C1—N1—C4—C3	−17.3 (2)	C12—N3—C9—C10	−65.5 (2)
C5—N1—C4—C3	169.93 (18)	C8—N3—C9—C11	−64.1 (2)
C2—C3—C4—N1	25.7 (2)	C12—N3—C9—C11	168.21 (18)
C1—N1—C5—C6	−94.6 (2)	C8—N3—C12—C13	−178.70 (17)
C4—N1—C5—C6	77.4 (2)	C9—N3—C12—C13	−51.3 (2)
N1—C5—C6—O2	−12.1 (3)	C8—N3—C12—C14	60.1 (2)
N1—C5—C6—N2	170.35 (18)	C9—N3—C12—C14	−172.46 (17)

### Hydrogen-bond geometry (Å, °)

**Table:**

*D*—H···*A*	*D*—H	H···*A*	*D*···*A*	*D*—H···*A*
N2—H2···O3	0.86 (3)	2.10 (3)	2.948 (2)	172 (2)
N3—H3···O4^i^	0.97 (3)	1.79 (3)	2.764 (2)	175 (2)
O6—H6···O1^ii^	0.90 (4)	1.68 (4)	2.559 (2)	167 (3)
C12—H12···O2^iii^	1.00	2.40	3.362 (3)	162
C13—H131···O4^iv^	0.98	2.59	3.559 (2)	171
C14—H143···O5	0.98	2.56	3.530 (2)	172

Symmetry codes: (i) −*x*+1, −*y*+1, −*z*+1; (ii) −*x*+1, −*y*+1, −*z*+2; (iii) *x*−1, *y*, *z*; (iv) −*x*, −*y*+1, −*z*+1.
